# Supplementary material for: The genome sequence of the Common Heath moth, Hypomecis atomaria (Linnaeus, 1758) (Lepidoptera: Geometridae)
Source: Wellcome Open Res. 2025 Nov 20;10:644. [Version 1] doi: 10.12688/wellcomeopenres.25218.1 (PMC12856257; doi:10.12688/wellcomeopenres.25218.1)
Supplement: Peer review history

# Reviewer report: Reviewer response for version 1

**DOI:** 10.21956/wellcomeopenres.27794.r143414

**Published:** 2026-01-29

**Authors:** Paulo Hofstatter

**Affiliations:** 1 University of Sao Paulo, São Paulo, State of São Paulo, Brazil

The author sequenced and assembled a *de novo* genomic dataset for the Lepidopteran *Hypomecis* *atomaria* from the British Isles. It is a high-quality assembly, whose methods are clear and sound. However, there is not a word about the choice for this organism: is it a pest? an important pollinator? is it endangered? Just sampling genomic biodiversity?

 It is not clear how chromosomes Z and W were identified (just 'size' is not enough). A simple synteny analysis comparing this assembly with another Lepidopteran dataset should be enough.

Are sufficient details of methods and materials provided to allow replication by others?

Yes

Is the rationale for creating the dataset(s) clearly described?

No

Are the datasets clearly presented in a useable and accessible format?

Yes

Are the protocols appropriate and is the work technically sound?

Yes

Reviewer Expertise:

genetics and evolution, bioinformatics, genomics.

I confirm that I have read this submission and believe that I have an appropriate level of expertise to confirm that it is of an acceptable scientific standard.

# Reviewer report: Reviewer response for version 1

**DOI:** 10.21956/wellcomeopenres.27794.r143416

**Published:** 2026-01-13

**Authors:** Cheng Sun

**Affiliations:** 1 Capital Normal University, Beijing, Beijing, China

The study successfully produced a high-quality genome sequence for the Common Heath moth, *Hypomecis atomaria*. The methods employed are well-described and robust, and the results appear to be reliable. Given the potential challenges in assigning sex chromosomes, I encourage the authors to provide additional details on the methods used to identify the Z and W chromosomes based on Hi-C data.

Are sufficient details of methods and materials provided to allow replication by others?

Yes

Is the rationale for creating the dataset(s) clearly described?

Yes

Are the datasets clearly presented in a useable and accessible format?

Yes

Are the protocols appropriate and is the work technically sound?

Yes

Reviewer Expertise:

insect genomics, genome evolution

I confirm that I have read this submission and believe that I have an appropriate level of expertise to confirm that it is of an acceptable scientific standard.
